# Queering Studies of Health and Bodily Experience: An Example From the Transgender Resilience and Health Study

**DOI:** 10.1002/ajpa.70183

**Published:** 2025-12-17

**Authors:** Dee Jolly, Jae A. Puckett, Cindi SturtzSreetharan, Sawyer E. Armstrong, Debra A. Hope, Richard Mocarski, Robert‐Paul Juster, L. Zachary DuBois

**Affiliations:** ^1^ Department of Anthropology University of Oregon Eugene Oregon USA; ^2^ Department of Psychology Michigan State University East Lansing Michigan USA; ^3^ School of Human Evolution and Social Change Arizona State University Tempe Arizona USA; ^4^ Department of Psychology University of Oregon Eugene Oregon USA; ^5^ Department of Psychology University of Nebraska‐Lincoln Lincoln Nebraska USA; ^6^ Division of Research and Innovation Partnerships Northern Illinois University DeKalb Illinois USA; ^7^ Department of Psychiatry and Addiction University of Montreal Montreal Quebec Canada

**Keywords:** embodiment, health inequities, minority stress, physical health, transgender health

## Abstract

**Objectives:**

Biological anthropologists have contributed significantly to our understanding of how lived experiences become embodied, affecting health. However, there has been less emphasis on bodily symptoms as an important aspect of health and well‐being impacted by lived experiences. Informed by queer and Black feminist approaches, we investigate the effects of stigma, stress, and support on transgender and nonbinary (TNB) people's bodily symptoms.

**Materials and Methods:**

Using baseline survey data from a longitudinal study of TNB people's health and resilience in the United States collected during fall 2019–spring 2020, we assessed the relationships between past year and lifetime enacted stigma, perceived stress, and bodily symptoms among 158 TNB people living in Michigan, Nebraska, Oregon, and Tennessee. Potential moderating factors were support from family and friends, resilience, gender identity, and racial identity.

**Results:**

Past year and lifetime enacted stigma and perceived stress were positively associated with bodily symptoms, whereas increased resilience and perceived support from family were associated with decreased bodily symptoms. However, chosen family support was not associated with bodily symptoms. The positive effects of resilience on health were independent of the negative effects of enacted stigma on bodily symptoms. Nonbinary people and TNB people of color experienced more severe bodily symptoms.

**Discussion:**

TNB people's lived experiences of the body reflect complex relationships between stigma, stress, resilience, and social supports, and vary by social position. Results emphasize the need to incorporate queer feminist perspectives of the body into biocultural conceptualizations and studies of health and embodiment.

## Summary

- Focusing on lived experiences of the body offers a way to center day‐to‐day lived experience as meaningful for health and well‐being.
- Lived experiences of stress and stigma become embodied, heightening symptom severity among TNB people in the United States.
- TNB people with higher resilience scores and greater family support experienced less severe bodily symptoms, independent of exposure to enacted stigma.

## 1 Introduction

Decades of anthropological research have furthered understandings of the ways lived experience and biology are intertwined and affect human health and well‐being, highlighting how our social worlds become embodied (Krieger 1999). Biocultural studies, in particular, have further linked aspects of lived experience to health, including risk and resilience (Panter‐Brick 2014), financial debt (Sweet et al. 2013, 2018), water insecurity (Brewis et al. 2019; Wutich 2020), weight stigma (Cullin 2023), racism (Gravlee 2009; Thayer and Kuzawa 2015), residential context (Nelson 2016), social status markers (McDade 2002), and gender‐based stigma (DuBois, Puckett, Jolly, et al. 2024; Puckett, Price, et al. 2024). But feminist and queer methodologies are less frequently *explicitly* employed in biological anthropology, despite the centrality of our work in contributing to knowledge about health inequities and social determinants of health.

Here we build on these contributions and draw on queer and feminist approaches to the body (Butler 2011; Malatino 2019; Rice et al. 2020) to expand assessments of health (as defined via diagnosable diseases) to include day‐to‐day bodily experiences. We do this through exploring how psychosocial stress and enacted stigma (i.e., experiences of discrimination, rejection, and victimization) contribute to variation in symptom severity. Bodily symptoms take a variety of forms, including experiences of pain, dizziness, nausea, or fatigue (Kroenke et al. 2002). Importantly, even when these bodily symptoms are not associated with clinical diagnoses or measured physiologic changes, they have significant and usually negative effects on people's daily lives (Dimsdale and Dantzer 2007). For example, pain is a common bodily symptom (Kroenke et al. 2002) that negatively impacts quality of life (Kawai et al. 2017). Chronic pain (i.e., pain lasting three or more months without an acute cause) is experienced by 50.2 million (~20%) adults in the United States (Yong et al. 2022). For ~4.1 million of these adults, chronic pain is borne from chronic experiences of *discrimination* (Brown et al. 2018). Furthermore, as reflected in the idea of biological normalcy (e.g., Wiley 2021), rigid reliance on clinical and diagnostic norms can also invisibilize and pathologize varied experiences, identities, bodies, and health statuses that do not reflect the majority of a population. In some cases, the reliance on clinical and diagnostic norms can also undermine inclusivity efforts; in this case, contributing to the invisibility of transgender and nonbinary (TNB) people (DuBois et al. 2021; Wiley 2021), who often experience barriers to care, diagnosis, and treatment (Berrian et al. 2025; Heng et al. 2018; Puckett et al. 2018; Tabaac et al. 2020). The impacts of inequity thus highlight the importance of studying how lived experiences affect bodily symptoms and their severity, particularly among those who experience stigma and marginalization.

### 1.1 Health Among Transgender and Nonbinary People

Most health‐focused research among TNB people has focused on mental health, partially due to cultural schemas that have treated people living outside the bounds of cissexist gender norms as deviant and disordered (i.e., via diagnoses of “gender identity disorder”) (Hoy‐Ellis 2023). Much less is known about physical health among TNB people, including chronic disease or aging, or the intertwinement of mental and physical health (Scheim et al. 2024). Biocultural anthropological approaches provide several recommendations and models to advance TNB health research by addressing gaps and developing more holistic and integrated approaches (DuBois et al. 2021). Relatedly, there are growing efforts to advance health research among TNB people through community‐engaged approaches (e.g., Aghi et al. 2022; Scheim et al. 2019, 2022). Because of the stark health inequities experienced by TNB communities (Dragon et al. 2017), much of the physical health research is focused on specific health outcomes related to HIV, gender‐affirming care, and cancer (Scheim et al. 2022). Less attention is paid to experiential and physiologic pathways that contribute to health, well‐being, and quality of life among TNB people.

Experiences of stigma and discrimination contribute to psychosocial stress, and these map onto well‐recognized physiological pathways through which certain health inequities develop and are exacerbated. Gender minority stress refers to stressors experienced *on top of* general stress experienced by TNB people in the context of cisnormativity and gender binarism (e.g., Matsuno et al. 2024; Testa et al. 2015). Gender minority stress can also impact TNB people's physical health (Christian et al. 2021; Juster et al. 2017). A growing body of research shows how gender minority stress and stigma become embodied via altered physiological function and dysregulation (DuBois 2012; DuBois et al. 2017; DuBois, Puckett, Jolly, et al. 2024; McQuillan et al. 2021; Rich et al. 2025), likely mediating the relationships between experiences of stigma and health inequities. However, less is known about bodily symptoms among TNB people or how stigma and gender minority stress affect symptom severity.

In response to calls for increasing recognition of the importance of stigma in biological anthropology (Brewis and Wutich 2020), here we contribute to the literature linking stigma and health among TNB people (for reviews, see Drabish and Theeke 2022; Reisner et al. 2016; White Hughto et al. 2015). Previous findings from this study indicated that lower levels of past year enacted stigma (i.e., discrimination, rejection, and victimization) mediated the association between legal gender affirmation (i.e., changing one's name and/or gender marker) and symptom severity (Puckett, Price, et al. 2024). This has important implications as symptoms are associated with a range of health conditions known to be more commonly experienced by TNB people relative to cisgender people, such as type II diabetes (Islam et al. 2022; Ogilvie and Patel 2018) and cardiovascular disease (Sawchuk and Olatunji 2011; van Zijverden et al. 2024). Because less is known about the cumulative effects of enacted stigma on health in this population, further exploration is warranted, as effects on health may accumulate over the lifespan (Krieger 2012). To move toward understanding aging and health among TNB people, it is also necessary to distinguish effects of gender minority stress from those of general life stress, as both are shown to have unique effects on mental health among older TNB adults (Hoy‐Ellis and Fredriksen‐Goldsen 2017).

It is also necessary to contribute nuance to our understanding of stigma and health among TNB people by describing potential differences in levels of exposure to stigma and stress and how embodied relationships among these and physical health may differ across different social identities and positions. For example, experiences of misgendering—a gender minority stressor associated with both mental and physical health (DuBois 2012; McLemore 2018)—varied based on gender identity and gender expression for individuals when they were wearing face masks (i.e., in response to COVID‐19). While masked, the frequency of misgendering was lessened among transgender women but increased among transgender men and nonbinary people (DuBois et al. 2023). Similar nuance is needed to better understand how the effects of gender minority stress on health may differ between TNB people of color (TNBPOC) and white TNB people. For example, lifelong exposure to racism may lead to increased expectations of discrimination while simultaneously being a source of resilience by making a person feel more prepared to cope with discrimination and stigma as a TNB person (Rood et al. 2016).

Another understudied area is how positive factors impact health and how these interact with negative lived experiences of stress and enacted stigma. Resilience, for example, may offset negative health effects of gender minority stress. Evidence of embodied resilience has been published from this study, where findings show a bolstered cortisol awakening response—a healthy adaptation to an otherwise dampened diurnal cortisol rhythm associated with elevated levels of anti‐TNB enacted stigma (DuBois, Puckett, Jolly, et al. 2024). Social support is another positive factor shown to buffer the health effects of gender minority stress (Trujillo et al. 2017). The type of social support (e.g., family, friends) has been shown to have different effects on TNB people's health, with family support being most impactful (Puckett et al. 2019). Importantly, many TNB people create chosen families (i.e., people considered family despite being unrelated), often in response to strained or absent relationships with their primary caregivers due to stigma and rejection (Weston 1997). Chosen family members are selected and interwoven into the tapestry of TNB people's lives, playing important roles in health through “mutual provision of care” (Jackson Levin et al. 2020, 16). Other studies have suggested that the effect of support from families of origin (i.e., a person's primary caregivers during key points of development) on health outweighs that of chosen family (Milton and Knutson 2023), creating a need to further explore how different kinds of familial supports and relationships affect health among TNB people.

### 1.2 Current Sociopolitical Context and TNB People in the United States

The current sociopolitical context of the United States heightens the need to investigate relations between gender minority stress and resilience and their impacts on TNB people's health. Hostility toward TNB communities has sharply escalated since 2020. More than 859 anti‐TNB bills were proposed across 49 states in the first 5 months of 2025, with 727 of those bills under active consideration (Trans Legislation Tracker 2025). Moreover, the Trump‐Vance administration issued an onslaught of anti‐TNB executive orders and presidential actions within the first 10 days of their administration, including bans on gender‐affirming healthcare and participation in sports, and erasure from all areas of the federal government, often under the guise of protecting cisgender women's safety and rights (The White House 2025a, 2025b, 2025c). This is similar to the “homonationalism” of the early 2000s that Puar (2007) describes, in which queer rights were used to justify exclusionist and nationalistic policies and attitudes toward people from South and Southwest Asia and North Africa. In the case of TNB people today, cisgender women's rights and concerns about safety are being politically co‐opted and weaponized against the rights of TNB people, while serving as a nationalistic rallying call to strengthen the power of the Trump‐Vance administration.

These bills and executive orders rely on pseudoscience and disorienting appeals to “common sense” to reinforce rigid, binary interpretations of sex and claim gender is indistinguishable from assigned sex at birth. This is in direct contradiction to the understanding of “sex” as a system of classification, based on a set of several biological traits (e.g., hormones, gametes, genitalia, gonads, chromosomes) that are assumed to align based on our social construction of what it means to be “male” or “female” (Bauer 2023; DuBois et al. 2025). Attempts to codify simplistic binary categories of sex serve to obfuscate meaningful human biological variation (DuBois and Shattuck‐Heidorn 2021; Springer et al. 2012) and enact structural and cultural acts of violence that promote stigma and legitimize direct acts of violence toward TNB people (Brightman et al. 2023; Galtung 1990). Predictably, political targeting and scapegoating of TNB people are associated with complex effects on the health and well‐being of TNB people (DuBois, Puckett, SturtzSreetharan, et al. 2024; Horne et al. 2022). The heightened political attacks also serve as a kind of social barometer indicative of a broader hostility toward TNB people. Perception of these hostile sociopolitical attitudes is itself linked to poorer mental health and altered stress pathways among TNB people (DuBois and Juster 2022; Puckett, Huit, et al. 2024). Consequently, it is increasingly important to understand the effects of enacted stigma on TNB people's health in the United States.

### 1.3 The Current Study

These analyses address the urgent need to nuance our understanding of embodied stigma and stress among TNB people by testing how past year and lifetime enacted stigma and general stress affect bodily symptoms. We also explore how resilience and social support may influence the associations between enacted stigma and bodily symptoms. Informed by Black feminist approaches (e.g., intersectionality theory) to understanding health (Bright et al. 2016; Collins 2019; Combahee River Collective 1977; Crenshaw 1989), we describe how embodied relationships of enacted stigma and stress differ for those with different social positions.

## 2 Methods and Materials

### 2.1 Participants and Study Protocol

A total of 158 TNB participants participated in the *Transgender Resilience and Health Study* (https://transresiliencestudy.com), which aimed to understand experiences and health among TNB people living in different sociopolitical contexts in the United States. Eligibility criteria required participants to (1) identify as transgender or nonbinary, (2) live in Michigan, Nebraska, Oregon, or Tennessee, and (3) be 19 years of age (age of consent in Nebraska) or older. A multi‐pronged recruitment approach included outreach to community organizations, distribution of physical flyers and advertisements at community events, and social media advertisements.

While sociopolitical climates for TNB people have shifted and arguably worsened since data were collected (2019–2021), the states included in this study deliberately reflected different sociopolitical climates in terms of state‐level policies and protections impacting TNB people. As detailed in prior publications from this study (see Puckett, Huit, et al. 2024), state selection was informed by data from the Human Rights Campaign and the Movement Advancement Project gender equality index, which aggregates data about state‐level legislative policies that either protect or target TNB people (Human Rights Campaign n.d.; Movement Advancement Project 2023). Sociopolitical climates at the state level at the time of site selection were rated as relatively supportive in Oregon, moderate in Michigan, and relatively hostile in Nebraska and Tennessee.

As part of the broader longitudinal study, all participants completed informed consent followed by a baseline assessment during fall 2019–spring 2020. Baseline assessments included a semi‐structured interview, anthropometric and minimally invasive biomarker measures, and electronically completed surveys. This visit took place at a location of the participants' choosing (e.g., local university, library, community center, or participants' homes). Participants received up to $250 via electronic gift cards for participating in the study, including completing baseline assessments, a year of monthly surveys, a final semi‐structured interview, and a final set of anthropometric and biomarker measures. This total payment included a $60 payment for those who completed the baseline study visit. The current analyses use data from the baseline surveys.

### 2.2 Measures

#### 2.2.1 Sociodemographics

A full list of sample demographics is available in Table 1.

**TABLE 1:** Sample demographics.

Characteristic	*N* (%)
Gender identity	
Transman/trans man	37 (23.4%)
Transwoman/trans woman	32 (20.3%)
Genderqueer	16 (10.1%)
Non‐binary	40 (25.3%)
Agender	3 (1.9%)
Androgyne	1 (0.6%)
Genderfluid	2 (1.3%)
Woman	9 (5.7%)
Man	6 (3.8%)
Bigender	2 (1.3%)
Not listed	9 (5.7%)
Missing	1 (0.6%)
Gender category	
Trans masculine	60 (38.2%)
Nonbinary/genderqueer	49 (31.2%)
Trans feminine	48 (30.6%)
Missing	1 (0.6%)
Sex assigned at birth	
Female	105 (66.5%)
Male	52 (32.9%)
Missing	1 (0.6%)
Race or ethnicity	
Black or African American	8 (5.1%)
American Indian or Alaskan Native	2 (1.3%)
Asian	6 (3.8%)
Latinx	6 (3.8%)
White	109 (69.0%)
Not listed	1 (0.6%)
Multiracial/multiethnic	26 (16.5%)
Sexual orientation (check all that apply)	
Bisexual	44 (27.8%)
Gay	23 (14.6%)
Lesbian	20 (12.7%)
Queer	83 (52.5%)
Asexual	16 (10.1%)
Pansexual	60 (38.0%)
Heterosexual/straight	8 (5.1%)
Not listed	8 (5.1%)
Education level	
High school graduate—high school diploma or equivalent (i.e., GED)	14 (8.9%)
Some college credit, but less than 1 year	8 (5.1%)
Technical or vocational school degree	5 (3.2%)
One or more years of college, no degree	42 (26.6%)
Associate's degree	18 (11.4%)
Bachelor's degree	52 (32.9%)
Master's degree	16 (10.1%)
Doctorate or professional degree (e.g., PhD, MD, JD, DDS)	2 (1.3%)
Graduate of a Certificate Program	1 (0.6%)
Individual income	
Less than $10,000	42 (27.2%)
10,000–19,999	38 (24.1%)
20,000–29,999	16 (10.1%)
30,000–39,999	12 (7.6%)
40,000–49,999	11 (7.0%)
50,000–59,999	14 (8.9%)
60,000–69,999	5 (3.2%)
70,000–79,999	8 (5.1%)
80,000–89,999	4 (2.5%)
90,000–99,999	0
More than $100,000	6 (3.8%)
Missing	1 (0.6%)
Age	
Gen Z (19–25 years)	60 (37.97%)
Millennial (26–39 years)	56 (35.4%)
Gen X (40–56 years)	31 (19.6%)
Boomer (57–70)	11 (7.0%)
Relationship status	
Single	61 (38.6%)
Dating	16 (10.1%)
In a relationship	45 (28.5%)
In multiple relationships	15 (9.5%)
Married	36 (22.8%)
Separated/divorced	9 (5.7%)

#### 2.2.2 Gender Identity

As we have previously detailed (DuBois, Puckett, Jolly, et al. 2024) and reflective of our approach to inclusive data collection methodologies (DuBois, Brewis, Burson, et al. 2024; DuBois and Shattuck‐Heidorn 2021), participants first self‐reported their gender identity by selecting from a list of 10 gender identity categories or providing a write‐in answer (see Table 1 for categories). Recognizing that collapsing categories is sometimes necessary to maximize analytic power, participants then self‐categorized into one of three categories (trans masculine, trans feminine, nonbinary/genderqueer). This approach improves accuracy in defining categories of experience, as opposed to researchers imposing these without participant input. For these analyses, we use the three categories of gender identity. This approach centers TNB perspectives, following best practices suggested by TNB people that assert an important distinction between gender identity and assigned sex at birth (Puckett et al. 2020).

#### 2.2.3 Racial Identity

We asked participants to report their racial identity through a check‐all‐that‐apply question. Options included Black or African American, American Indian or Alaska Native, Native Hawaiian or other Pacific Islander, Asian, Latino/a/x, white, and a write‐in option. Participants who identified with two or more racial categories were grouped as multiracial/multiethnic. For these analyses, due to small sample sizes in all racial groups besides those identified as white, two categories were used to reflect participants as being a transgender or nonbinary person of color (TNBPOC; *N* = 49, 31.0%) or as white TNB people (*N* = 109, 69.0%).

#### 2.2.4 Patient Health Questionnaire‐15

The Patient Health Questionnaire‐15 (PHQ‐15) is a self‐administered scale that assesses the severity of 15 bodily symptoms, such as pain, fatigue, and nausea (Kroenke et al. 2002). Participants were asked to rate how much each symptom has bothered them in the last 4 weeks. Response options were: 0 (“not bothered at all”), 1 (“bothered a little”), and 2 (“bothered a lot”). A sum score was calculated, ranging from a possible 0 to 30. Clinical scoring cutoffs are minimal (0–4), low (5–9), medium (10–14), and high (15–30). Cronbach's alpha was 0.78.

#### 2.2.5 Gender Minority Stress and Resilience Measure

The Gender Minority Stress and Resilience Measure (GMSR) (Testa et al. 2015) was used to measure TNB‐related enacted stigma (i.e., discrimination, rejection, victimization subscales). Using a check‐all format, participants indicated whether they experienced each item “never,” “yes, before age 18,” “yes, after age 18,” and “yes, in the past year.” *Past year enacted stigma* was calculated based on a sum score of the number of items endorsed in the past year. *Lifetime enacted stigma* was calculated based on a sum score of items where a participant checked any option other than “never.” Past year and lifetime enacted stigma ranged from a possible 0 to 17. Cronbach's alpha was respectively 0.82 and 0.76.

#### 2.2.6 Perceived Stress Scale

The Perceived Stress Scale measures general life stress (Cohen et al. 1983) over the past month. Responses for each item ranged from 0 (“never”) to 4 (“very often”). A sum score was calculated, ranging from a potential 0 to 40. Cronbach's alpha was 0.91.

#### 2.2.7 Brief Resilience Scale

The Brief Resilience Scale measures a person's ability to recover from stress (Smith et al. 2008). Responses for each item ranged from 1 (“strongly disagree”) to 5 (“strongly agree”), and an average score was calculated. Cronbach's alpha was 0.91.

#### 2.2.8 Multidimensional Scale of Perceived Social Support

The Multidimensional Scale of Perceived Social Support (MSPSS) measures social support from different sources of support (e.g., friends, family) (Zimet et al. 1988). Instructions guided participants to consider “family” as “family of origin or the family you grew up with.” Responses for each item ranged from 1 (“very strongly disagree”) to 7 (“very strongly agree”). An average score was calculated for each subscale (i.e., family, friends), ranging from a possible 1 to 7 for each subscale. Cronbach's alpha was respectively 0.92 and 0.89.

#### 2.2.9 Chosen Family

After completing the MSPSS, participants were asked if they have people in their lives that they view as family that are not related to them. Those who reported having a chosen family (*N* = 120, 75.9%) completed an investigator‐modified version of the MSPSS, containing the 4 items of the family subscale of the MSPSS modified to read “chosen family” instead of “family.” An average score was then calculated. Cronbach's alpha was 0.94.

### 2.3 Data Treatment and Statistical Analysis

Data were first checked for normality. The friend and chosen family support variables were cube‐transformed to reduce skew and kurtosis to acceptable levels (i.e., +/− 1 skew and +/− 4 kurtosis). Analyses were conducted with the transformed and untransformed variables, and results were the same, regardless of data transformation, suggesting that these variables were not skewed enough to affect the results. To improve interpretability, we report findings using the untransformed variables.

Pearson correlation was used to identify salient factors associated with bodily symptoms among TNB people. ANOVA and *t*‐tests were respectively used to assess sociodemographic‐based differences (i.e., gender identity, racial identity) in bodily symptoms. Following ANOVA, Tukey's test for honest differences was used to assess true differences between gender identity categories.

Moderation analyses were performed to examine how resilience and social supports interact with lived experiences of stress and stigma on bodily symptoms. Main effects indicate impacts of social support and resilience on symptom severity after adjusting for enacted stigma and general stress. Interaction effects assess if resilience and social support buffer the effects of stigma and stress on symptom severity. Only factors of resilience and support identified as significant during initial analyses were tested in moderation analyses.

Moderation analyses also assessed if associations between enacted stigma, general stress, and symptom severity significantly differed by gender or racial identity. Here, “main effects” from the gender or racial identity variables indicate gender or racial identity‐based differences in bodily symptom severity beyond the effects of enacted stigma and perceived stress, whereas the interaction term indicates potential differences in the association between the identity groups.

To identify potential covariates, we first tested associations between symptom severity and potential covariates through bivariate correlations and ANOVA analyses. Age, chronic disease (*N* = 92, 58.2%), medication usage (*N* = 111, 70.7%), income, and relationship status were all tested as potential covariates. Age (*r* = −0.08, *p* = 0.34), income (*F*(9, 143) = 1.48, *p* = 0.16), and relationship status were not associated with bodily symptoms (*F*(6,147) = 0.70, *p* = 0.65). Having a chronic disease (*r* = 0.32, *p* < 0.001) and medication usage (*r* = 0.33, *p* < 0.001) were associated with bodily symptoms and retained in the analytic models.

Continuous variables were mean‐centered prior to moderation analyses. In response to critiques of the utility of *p*‐values in detecting meaningful differences (Amrhein et al. 2019; Wasserstein and Lazar 2016), confidence intervals and marginal plots were used to interpret model effects rather than relying on *p*‐values alone. Mean values and values one standard deviation away from the mean were used to test potential differences in relationships at low, average, and high levels of each continuous factor. Post hoc power analyses were used to help guide interpretation of findings from the moderation analyses. Analyses were conducted in Stata 16 (StataCorp 2019), except post hoc power analyses conducted in G*Power 3 (Faul et al. 2007). The data from this study are not publicly available due to privacy and ethical restrictions.

## 3 Results

### 3.1 What Factors Are Associated With Bodily Symptom Severity?

Nearly half of our sample (*N* = 77, 48.7%) reported having “medium” or “high” symptom severity (following Kroenke et al. 2002). Symptom severity was positively associated with both past year and lifetime enacted stigma and general stress (Table 2). Social support from family and resilience were both negatively associated with symptom severity. Neither support from friends nor support from *chosen* family was significantly associated with symptom severity.

**TABLE 2:** Associations between bodily symptoms, stigma, stress, resilience, and different forms of social supports.

	1.	2.	3.	4.	5.	6.	7.
1. Bodily symptoms	—						
2. Past year enacted stigma	0.29***	—					
3. Lifetime enacted stigma	0.21*	0.47***	—				
4. Perceived stress	0.53***	0.30***	0.14^†^	—			
5. Resilience	−0.35***	−0.18*	−0.07	−0.63***	—		
6. Family of origin support	−0.19*	−0.18*	−0.25**	−0.27**	0.20*	—	
7. Friend support	−0.02	−0.11	−0.21*	−0.15^†^	0.11	0.25**	—
8. Chosen family support	0.13	−0.04	0.03	0.11	−0.11	−0.07	0.22*

*Note:* ^†^ *p* < 0.10, *indicates *p* < 0.05, **indicates *p* < 0.01, ***indicates *p* < 0.001.

### 3.2 Do Bodily Symptom Severity or Reported Lived Experiences Differ by Sociodemographic Characteristics?

Symptom severity differed significantly by gender identity; nonbinary participants experienced more severe symptoms than both trans feminine and trans masculine participants (Table 3). We did not find significant differences between gender identity groups in regard to enacted stigma, general stress, resilience, and family or friend support. TNBPOC reported marginally more severe bodily symptoms than white TNB people, but there were no significant differences in exposure to stress and stigma, resilience, or support from family, friends, or chosen family.

**TABLE 3:** Variation in bodily symptoms and all tested factors by sociodemographic characteristics.

	Bodily symptoms	Past year enacted stigma	Lifetime enacted stigma	Perceived stress	Resilience	Family support	Friend support	Chosen family support
	Mean (standard deviation)							
Total sample	9.46 (4.82)	3.93 (3.04)	8.43 (3.92)	20.54 (7.35)	2.99 (0.96)	3.61 (1.83)	5.75 (1.14)	5.80 (1.42)
Gender identity								
Trans masculine	8.76 (4.10)_‡_	3.36 (2.98)	8.28 (3.55)	20.32 (7.84)	3.04 (1.03)	3.97 (1.81)	5.81 (0.95)	5.79 (1.48)
Nonbinary/genderqueer	11.80 (4.67)	4.13 (3.15)	7.92 (4.23)	21.63 (6.79)	2.91 (0.86)	3.46 (1.78)	5.83 (1.12)	6.20 (0.93)
Trans feminine	7.85 (4.90)_‡_	4.42 (2.97)	9.08 (4.03)	19.46 (7.15)	3.02 (0.96)	3.33 (1.88)	5.61 (1.37)	5.44 (1.64)_‡_
*p*‐value	< 0.001	0.172	0.339	0.339	0.769	0.156	0.582	0.081
Race and ethnicity								
TNBPOC	10.46 (5.34)	3.44 (0.42)	8.62 (0.60)	21.69 (1.03)	2.81 (0.13)	3.61 (0.27)	5.61 (0.17)	5.72 (1.46)
White TNB	9.01 (4.52)	4.15 (0.30)	8.35 (0.37)	20.04 (0.71)	3.07 (0.09)	3.60 (0.18)	5.81 (0.11)	5.83 (1.41)
*p*‐value	0.084	0.178	0.702	0.196	0.124	0.996	0.297	0.709

*Note:* ‡ indicates a statistically significant difference relative to nonbinary people; *p*‐values are from ANOVA analyses.

### 3.3 Do Social Support and Resilience Buffer the Effects of Perceived Stress and Enacted Stigma?

**FIGURE 1:**
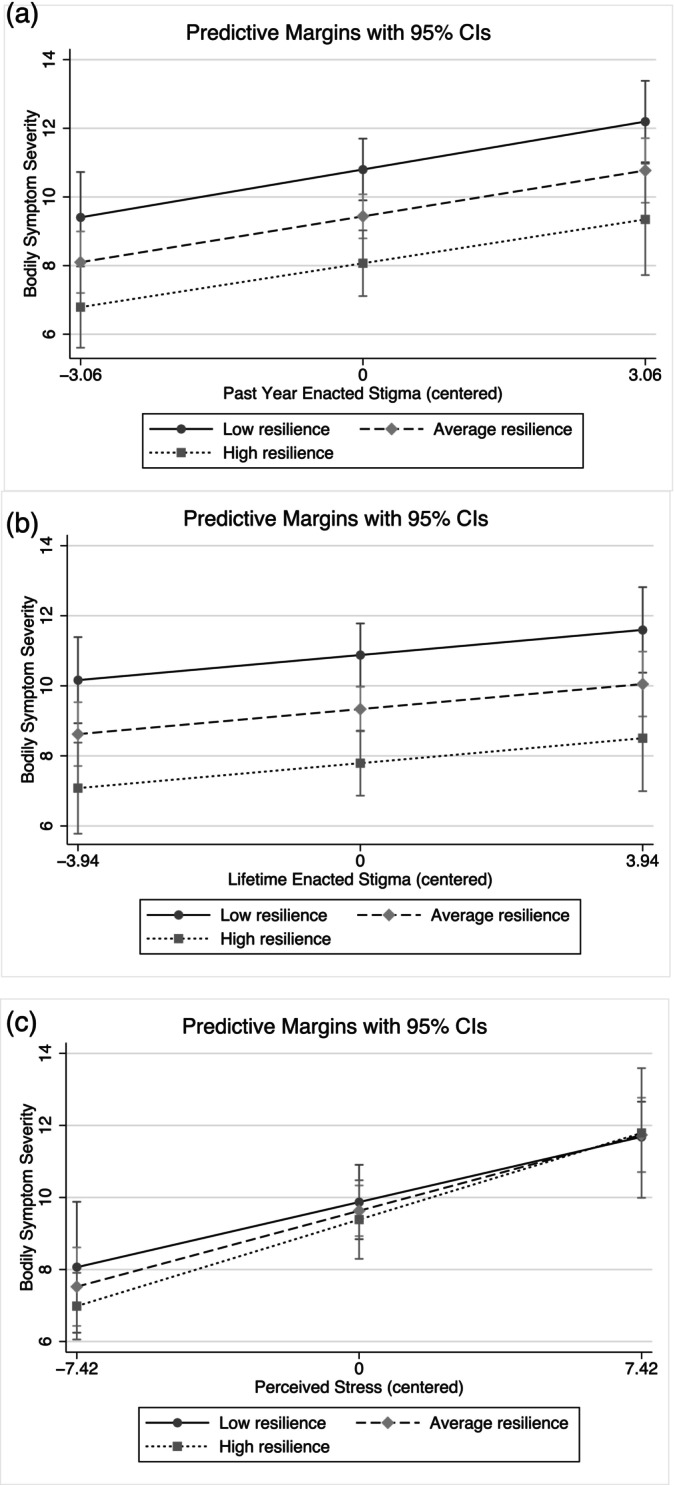
Predicted relationships between stress and bodily symptoms moderated by resilience. (a–c) Represent past year enacted stigma, lifetime enacted stigma, and perceived stress models, respectively.

**FIGURE 2:**
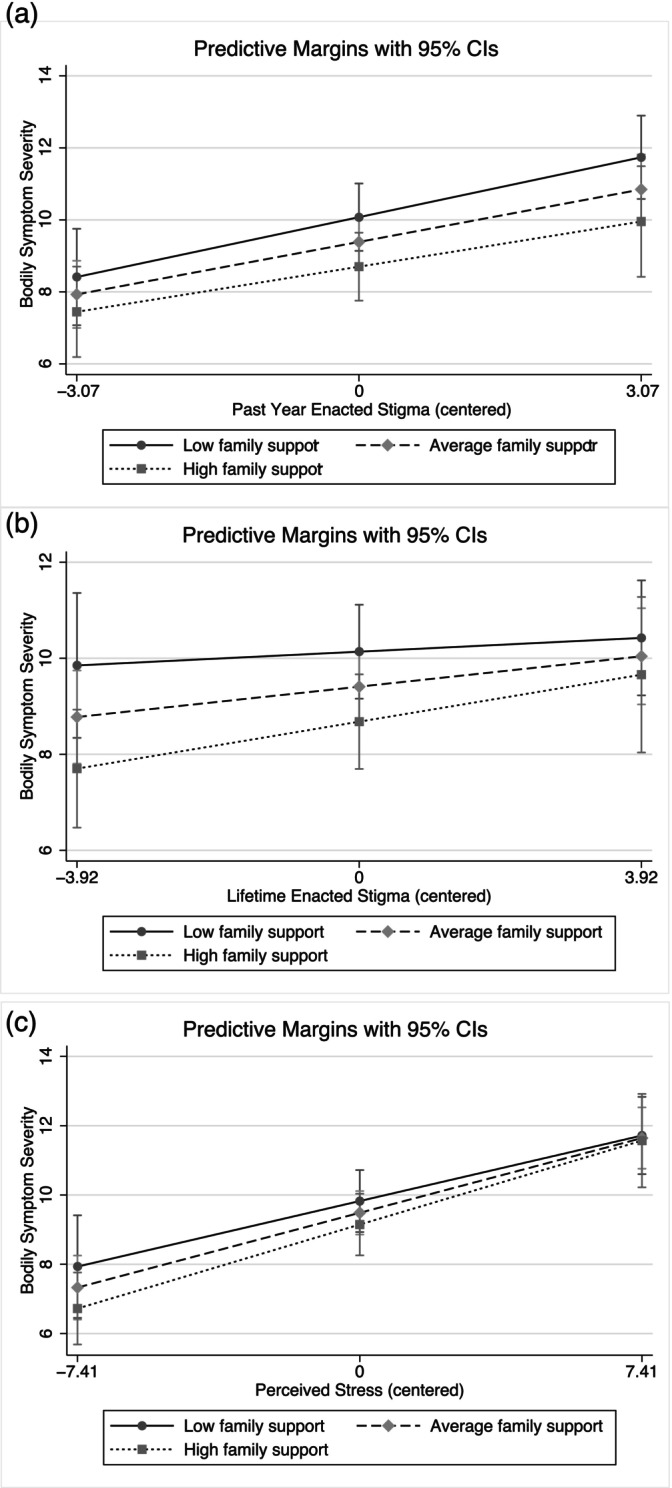
Predicted relationships between stress and bodily symptoms moderated by perceived family support. (a–c) Represent past year enacted stigma, lifetime enacted stigma, and perceived stress models, respectively.

Resilience and family support were not significant moderators of the associations between past year enacted stigma, lifetime enacted stigma, or general stress and symptom severity (Table 4 and Table 5). Resilience, however, did have effects independent of enacted stigma on the severity of bodily symptoms. In other words, at higher levels of resilience, predicted symptom severity was lower, even after adjusting for the effects of enacted stigma. However, no buffering effect was detected as the association between enacted stigma and symptom severity was not weaker at higher levels of resilience (see slopes in Figure 1). Likewise, greater family support was significantly associated with less severe symptoms but did not buffer the effect of enacted stigma on symptom severity (Table 5, Figure 2). There was a significant positive association between enacted stigma and symptom severity regardless of levels of resilience or family support (Tables 4 and 5).

**TABLE 4:** Relationships between stress and bodily symptoms moderated by resilience.

	Bodily symptoms					
	Past year enacted stigma		Lifetime enacted stigma		Perceived stress	
	B	95% CI	B	95% CI	B	95% CI
Past year enacted stigma	0.437***	0.224, 0.651				
Lifetime enacted stigma			0.181*	0.014, 0.349		
Perceived stress					0.283***	0.176, 0.391
Resilience	−1.438***	−2.143, −0.733	−1.624***	−2.317, −0.932	−0.253	−1.092, 0.586
Resilience × past year enacted stigma	−0.020	−0.264, 0.224				
Resilience × lifetime enacted stigma			−0.001	−0.183, 0.182		
Resilience × perceived stress					0.042	−0.039, 0.123
Has a chronic disease	3.151***	1.849, 4.452	2.683***	1.360, 4.005	2.572***	1.311, 3.833
Taking medication	2.596**	1.149, 4.043	2.334**	0.888, 3.780	2.060**	0.679, 3.442
Model fit	*R* ^2^ = 0.361 Δ*R* ^2^ = < 0.001 *F*(5,147) = 16.63, *p* < 0.001		*R* ^2^ = 0.310 Δ*R* ^2^ < 0.001 *F*(5, 146) = 13.14, *p* < 0.001		*R* ^2^ = 0.408 Δ*R* ^2^ = 0.004 *F*(5, 147) = 20.28, *p* < 0.001	

*Note:* ^†^indicates *p* < 0.10, *indicates *p* < 0.05, **indicates *p* < 0.01, ***indicates *p* < 0.001. Change in *R* ^2^ represents additional variance accounted for by the interaction effects.

**TABLE 5:** Relationships between stress and bodily symptoms moderated by perceived family support.

	Bodily symptoms					
	Past year enacted stigma		Lifetime enacted stigma		Perceived stress	
	B	95% CI	B	95% CI	B	95% CI
Past year enacted stigma	0.475***	0.252, 0.698				
Lifetime enacted stigma			0.162^†^	−0.017, 0.341		
Perceived stress					0.291***	0.203, 0.379
Perceived family support	−0.375*	−0.739, −0.011	−0.398*	−0.776, −0.020	−0.185	−0.531, 0.161
Perceived family support × past year enacted stigma	−0.036	−0.151, 0.079				
Perceived family support × lifetime enacted stigma			0.048	−0.051, 0.146		
Perceived family support × perceived stress					0.020	−0.025, 0.064
Has a chronic disease	2.766***	1.422, 4.110	2.335**	0.947, 3.723	2.504***	1.256, 3.753
Taking medication	3.221***	1.759, 4.683	2.953***	1.457, 4.450	2.144**	0.762, 3.526
Model fit	*R* ^2^ = 0.308 Δ*R* ^2^ = 0.002 *F*(5, 146) = 13.02, *p* < 0.001		*R* ^2^ = 0.227 Δ*R* ^2^ = 0.005 *F*(5, 145) = 8.51, *p* < 0.001		*R* ^2^ = 0.403 Δ*R* ^2^ = 0.003 *F*(5, 146) = 19.71, *p* < 0.001	

*Note:* ^†^indicates *p* < 0.10, *indicates *p* < 0.05, **indicates *p* < 0.01, ***indicates *p* < 0.001. Change in R^2^ represents additional variance accounted for by the interaction effects.

Neither resilience nor family support was significantly associated with symptom severity after adjusting for general stress (Tables 4 and 5). There was not a si gnificant difference in the associations between general stress and symptom severity at differing levels of resilience or family support (Figures 1 and 2). Given our small sample size and being underpowered to detect significant moderating effects, these results should be interpreted with caution.

### 3.4 Do the Associations Between Symptom Severity and Enacted Stigma and General Stress Differ Between Gender and Racial Identity Groups?

**FIGURE 3:**
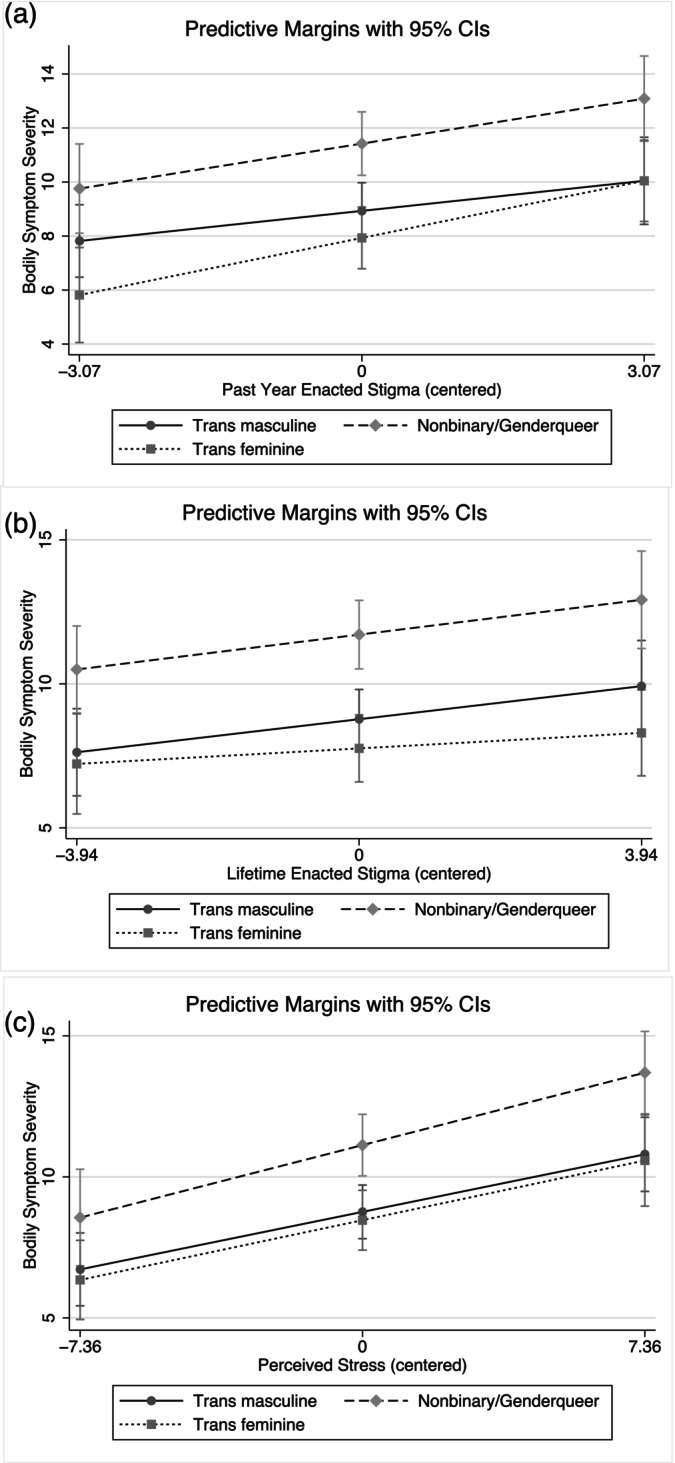
Predicted relationships between stress and bodily symptoms moderated by gender identity. (a–c) Represent past year enacted stigma, lifetime enacted stigma, and perceived stress models, respectively.

Nuancing the simple group‐based differences in symptom severity among people of different gender identities reported in Table 3, gender identity did not moderate the association between past year and lifetime enacted stigma, general stress, and symptom severity (Table 6). As such, the association between enacted stigma and perceived stress did not significantly differ among people of varying gender identities (Table 6, Figure 3). However, the plots in Figure 3 suggest a potential trend toward gender identity‐based differences in the association between enacted stigma and symptom severity.

**TABLE 6:** Relationships between stress and bodily symptoms moderated by gender identity.

	Bodily symptoms					
	Past year enacted stigma		Lifetime enacted stigma		Perceived stress	
	B	95% CI	B	95% CI	B	95% CI
Past year enacted stigma	0.542**	0.181, 0.904				
Lifetime enacted stigma			0.307*	0.034, 0.580		
Perceived stress					0.349***	0.192, 0.506
Trans masculine identity	−2.487**	−4.073, −0.901	−2.935***	−4.524, −1.346	−2.365**	−3.828, −0.901
Trans feminine identity	−3.494***	−5.155, −1.834	−3.946***	−5.634, −2.258	−2.660***	−4.200, −1.120
Trans masculine × past year enacted stigma	−0.180	−0.681, 0.322				
Trans feminine × past year enacted stigma	0.146	−0.380, 0.673				
Trans masculine × lifetime enacted stigma			−0.015	−0.413, 0.382		
Trans feminine × lifetime enacted stigma			−0.170	−0.566, 0.226		
Trans masculine × perceived stress					−0.072	−0.269, 0.126
Trans feminine × perceived stress					−0.061	−0.276, 0.153
Has a chronic disease	2.868***	1.558, 4.179	2.231**	0.901, 3.560	2.538***	1.333, 3.744
Taking medication	2.346**	0.885, 3.806	1.935*	0.462, 3.409	1.366^†^	−0.003, 2.736
Model fit	*R* ^2^ = 0.368 Δ*R* ^2^ = 0.007 *F*(7, 144) = 11.98, *p* < 0.001		*R* ^2^ = 0.316 Δ*R* ^2^ = 0.004 *F*(7, 143) = 9.44, *p* < 0.001		*R* ^2^ = 0.451 Δ*R* ^2^ = 0.002 *F*(7, 145) = 17.05, *p* < 0.001	

*Note:* ^†^indicates *p* < 0.10, *indicates *p* < 0.05, **indicates *p* < 0.01, ***indicates *p* < 0.001. People with a nonbinary identity were selected as the reference group due to being the group with the highest bodily symptoms. Change in *R* ^2^ represents additional variance accounted for by the interaction effects.

**FIGURE 4:**
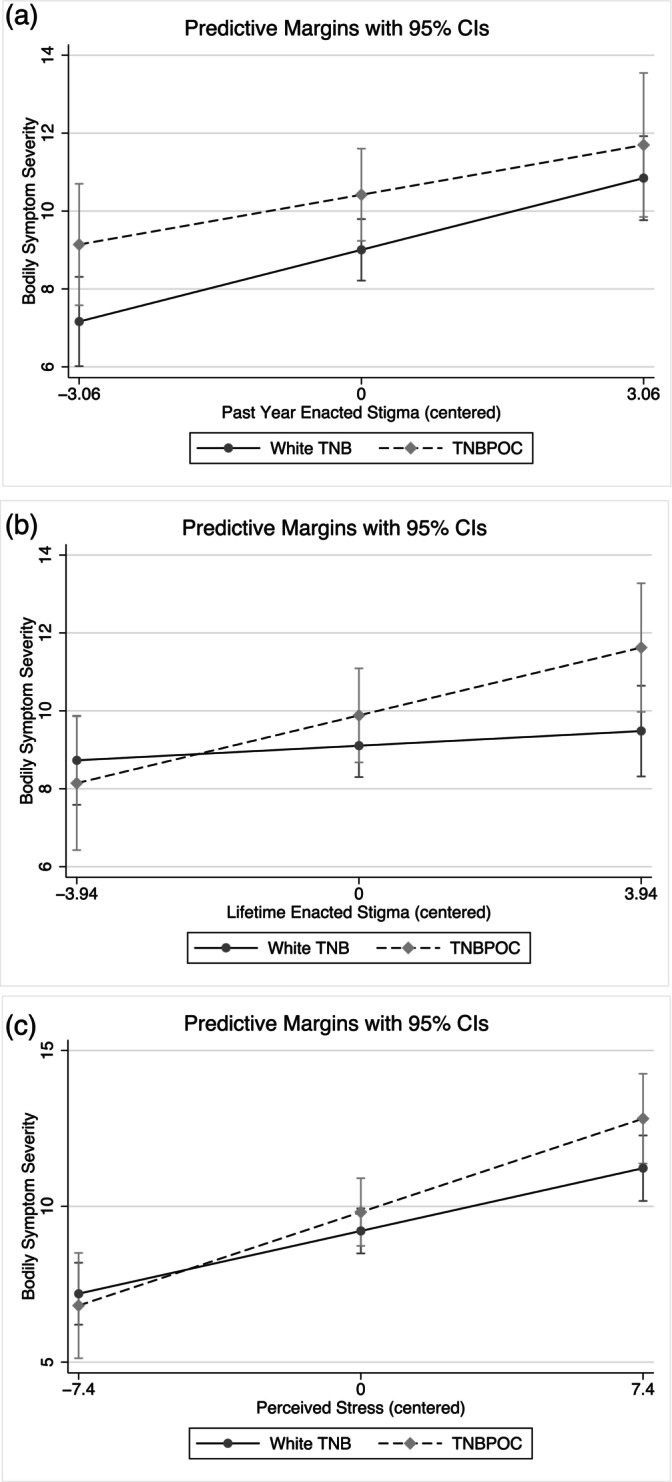
Predicted relationships between stress and bodily symptoms moderated by race and ethnicity. (a–c) Represent past year enacted stigma, lifetime enacted stigma, and perceived stress models, respectively.

For past year enacted stigma, TNBPOC participants experienced significantly more severe symptoms compared to white TNB participants when controlling for the effects of past year enacted stigma in our initial analyses without covariates. However, there was no longer a significant association between TNBPOC identity and bodily symptom severity in any of our analyses after adjusting for chronic disease and medication usage (Table 7). Unlike past year enacted stigma, having a TNBPOC identity did moderate the relationship between lifetime enacted stigma and symptom severity, although the interaction was no longer significant in our adjusted analyses following the addition of chronic disease (Table 7). Even so, the plots in Figure 4 continue to suggest a trend toward a stronger relationship between lifetime enacted stigma and symptom severity for TNBPOC compared to white TNB people. Furthermore, calculated conditional effects indicate a significant association between lifetime enacted stigma and symptom severity that was present among TNBPOC but not white TNB people (*B*_*TNBPOC*_ = 0.44, *p* < 0.01, 95% CI = 0.14, 0.74; *B*_*White TNB*_ = 0.10, *p* = 0.37, 95% CI = −0.11, 0.30). Having a TNBPOC identity did not significantly moderate the effects of past year enacted stigma or perceived stress on symptom severity (Table 7, Figure 4).

**TABLE 7:** Relationships between stress and bodily symptoms moderated by race and ethnicity.

	Bodily symptoms					
	Past year enacted stigma		Lifetime enacted stigma		Perceived stress	
	B	95% CI	B	95% CI	B	95% CI
Past year enacted stigma	0.418*	0.014, 0.821				
Lifetime enacted stigma			0.442**	0.200, 0.842		
Perceived stress					0.405***	0.249, 0.579
White TNB identity	−1.417^†^	−2.840, 0.006	−0.781	−2.233, 0.672	−0.610	−1.915, 0.694
White TNB identity × past year enacted stigma	0.184	−0.295, 0.663				
White TNB identity × lifetime enacted stigma			−0.347^†^	−0.710, 0.016		
White TNB identity × perceived stress					−0.133	−0.314, 0.048
Has a chronic disease	2.807***	1.462, 4.152	2.185**	0.797, 3.574	2.562***	1.322, 3.803
Taking medication	3.201***	1.726, 4.676	2.881***	1.382, 4.379	2.006**	0.640, 3.371
Model fit	*R* ^2^ = 0.311 Δ*R* ^2^ = 0.003 *F*(5, 147) = 13.30, *p* < 0.001		*R* ^2^ = 0.232 Δ*R* ^2^ = 0.019 *F*(5, 146) = 8.80, *p* < 0.001		*R* ^2^ = 0.414 Δ*R* ^2^ = 0.008 *F*(5, 148) = 20.93, *p* < 0.001	

*Note:* ^†^indicates *p* < 0.10, *indicates *p* < 0.05, **indicates *p* < 0.01, ***indicates *p* < 0.001. People with a TNBPOC identity were selected as the reference group due to being the group with the highest bodily symptoms. Change in *R* ^2^ represents additional variance accounted for by the interaction effects.

## 4 Discussion

Our results illustrate the complex ways that enacted stigma affects the symptom severity among TNB people. The heightened severity of symptoms experienced by almost half our sample may reflect exposure to enacted stigma and stress, as has been found in similar studies among communities exposed to stigma and inequity (e.g., body weight, HIV status) (Prunty et al. 2023; Rueda et al. 2016). These findings also complement the growing body of literature linking gender minority stress and stigma to stress‐linked physiology with long‐term implications for health (DuBois 2012; DuBois et al. 2017; DuBois, Puckett, Jolly, et al. 2024; McQuillan et al. 2021).

### 4.1 Implications for Chronic Disease and Aging Among TNB People

Nearly one‐half of our sample reported having moderate to severe symptoms. Given the potential downstream effects of bodily symptoms contributing to the development or worsening of chronic disease (Ogilvie and Patel 2018; Sawchuk and Olatunji 2011), our results point to an increased risk for developing or worsening chronic disease over one's lifetime due to the negative effects of enacted stigma and general stress. The complex relationships among enacted stigma, symptom severity, and chronic disease may also partially explain why higher rates of chronic disease are found among TNB people in the United States compared to cisgender people (Dragon et al. 2017) and may also impact well‐being among TNB people by reducing quality of life (Hinz et al. 2017). Reducing exposure to stigma and stress would facilitate health, well‐being, and healthy aging (i.e., aging in a way that allows a person “to be and do what they value throughout their lives;” World Health Organization 2020).

### 4.2 Enacted Stigma Versus General Stress

Aligned with other studies among TNB people (Hoy‐Ellis and Fredriksen‐Goldsen 2017), our findings suggest that both gender minority stress, including enacted stigma as measured in this study, and general stress interact with experiences of resilience and support in distinct ways to impact TNB people's experiences of the body. Though some studies suggest associations between general stress (i.e., PSS), health, and health behaviors among TNB people (McElroy et al. 2016; Sivaranjani et al. 2019; Xu et al. 2023), general stress measures may not sufficiently capture experiences of gender minority stress found to have embodied effects and implications for health (DuBois 2012; DuBois et al. 2017; DuBois and Juster 2022). This highlights the need for researchers to develop and utilize community‐based measures to assess experiences beyond general stress. Indeed, since collecting data for this study, several new scales have been developed and validated that capture resilience (Puckett, Matsuno, et al. 2025) and several novel scales assessing varied forms of gender marginalization and stigma (Puckett, DuBois, et al. 2025). Ideally, future data collection and community‐based research can further nuance our understanding of how exposure to different types of stigma and stressors impact health among TNB people.

The negative association between general stress and the severity of symptoms in our sample could also reflect that this measure is capturing the general and pervasive stress of anti‐TNB stigma in society. Others have found uncertainty affects health (Greco and Roger 2003; Peters et al. 2017). TNB people may also experience this kind of stress when bracing for legislative decisions impacting their bodily autonomy, access to medical care, and ability to participate in everyday aspects of life (e.g., using gender‐segregated public restrooms). In fact, the scale specifically asks if participants feel that they “were unable to control the important things in [their lives]”—a reasonable response to anti‐TNB stigma.

### 4.3 Resilience May Reduce Symptom Severity

In addition to enhancing our understanding of embodied stigma and gender minority stress, our results suggest that resilience, defined as the ability to recover from stress, can have powerful benefits for TNB people's health and lived experiences of the body in the context of enacted stigma. These findings support the need for affirmative psychological interventions to promote resilience among TNB people as an important and beneficial intervention to improve the health of TNB people (Expósito‐Campos et al. 2023). However, resilience did not moderate the relationship between enacted stigma and symptom severity, consistent with the “direct effects” model in which resilience offers benefits for health even outside of the context of stress rather than a “buffering effect” model (Cohen and Wills 1985). This finding echoes research conducted with other marginalized groups that suggest that resilience factors themselves do not moderate the effects of enacted stigma on physical health (Earnshaw et al. 2015). Furthermore, results showed that the negative effects of enacted stigma on symptom severity persisted after accounting for levels of resilience. These findings suggest that resilience alone may not entirely mitigate the effects of structural inequity, or the harm of a hostile, stigmatizing climate that creates, enables, and encourages discrimination, rejection, and violence toward TNB people. This underscores the need to reduce overall exposure to enacted stigma and stress. However, it is also possible that we lacked sufficient power due to the relatively small sample size to detect a meaningful buffering effect, especially given the small effect size of the interaction term.

### 4.4 The Source of Social Support Matters

Our analyses suggest that increased family support, defined broadly in the scale as “family of origin or the family you grew up with” is linked to less severe symptoms, independent of exposure to enacted stigma. While the MSPSS family subscale asks broadly about “family support” from the “family of origin,” the scale does not define who specifically belongs to that family. Because many TNB people also have chosen families who enrich their lives (Jackson Levin et al. 2020; Weston 1997), we also asked specifically about support from “chosen family.” However, aligned with other literature studying how family support affects health among TNB people (Milton and Knutson 2023; Puckett et al. 2019), support from friends or chosen family members was not associated with symptom severity, highlighting the importance of support from primary caregivers (i.e., “families of origin”) in shaping TNB people's health. Given that roughly one‐third of TNB people report having family members not speak to them or ending relationships because of their TNB identity (Marquez‐Velarde et al. 2023), a lack of familial support may present a common chronic stressor affecting health among TNB people. As a chronic stressor, estrangement from one's primary caregivers (e.g., familial rejection) may lead to cumulative ‘wear and tear’ on the body and (i.e., increased allostatic load), which leads to negative health outcomes and bodily symptoms (Juster et al. 2010). For example, Wickrama et al. (2023) found evidence that familial rejection in adolescence was associated with both increased allostatic load and development of chronic pain in young adulthood; pain is a common bodily symptom included in this study. This also implies that developing interventions for family members to accept, understand, and support their TNB family members may be protective for TNB people's health.

### 4.5 Gender Identity‐Based Differences in the Relationship Between Enacted Stigma and Health

We found gender‐based differences in the severity of symptoms, such that nonbinary people experienced more severe symptoms than participants who identified as trans feminine and trans masculine, even after accounting for the effects of enacted stigma and general stress. These results align with other research indicating differences in health among TNB people of different gender identities (Cicero et al. 2020; Flentje et al. 2022; Streed et al. 2018). Moreover, there were no differences by gender identity in exposure to stigma and stress. However, aligned with research suggesting differences among TNB people of varying gender identities in how different types of minority stressors are embodied (Flentje et al. 2022), our moderation plots indicated a trend toward differences in associations that we may have been underpowered to detect. We interpret this difference in association as potential gender differences in embodied relationships among stigma, stress, and health. Further research is needed to elucidate the mechanisms producing differences in health among TNB people of different gender identities.

The gendered differences in symptom severity observed in our sample may be attributable to gender identity‐related differences in *how* gender binarism (i.e., the belief that there are only two genders) and cisgenderism are experienced, which may not have been captured by the GMSR measure used in this study. Gender binarism affects all TNB people through social pressures to conform to gender/sex‐based norms (Dietert and Dentice 2013). This is in part due to implicit ties between gender binarism and rigid enforcement of gender/sex categories and norms that privilege cisgender heteronormative gender expression and identities over TNB gender expression and identities (i.e., cisgenderism). TNB people of different gender identities experience gender binarism differently, contributing to different effects on health (DuBois, Puckett, Jolly, et al. 2024). For example, nonbinary people occupy a social identity constructed outside the norms of binary categories of gender/sex and thus have unique experiences of gender binarism as a minority stressor (Matsuno et al. 2024). This minority stressor of cisgenderism also applies more broadly to TNB people in general (Puckett et al. 2023). Likewise, trans feminine people occupy a social position that challenges cisgenderist and patriarchal norms, leading to unique experiences of transmisogyny (Serano 2016). These differences in *how* gender binarism and cisgenderism are experienced and embodied may lead to the observed differences in bodily symptoms.

### 4.6 Racial Identity‐Based Differences in the Relationship Between Enacted Stigma and Symptom Severity

As with gender identity, we found no significant differences in overall exposure to enacted stigma and general stress between TNBPOC and white TNB participants. However, prior to the addition of covariates, we found that there were racial identity‐based differences in the association between lifetime enacted stigma and symptom severity, suggesting that the more severe bodily symptoms experienced by TNBPOC reflect differences in how stigma and stress are embodied over the lifetime. While this difference fell to a trending level following the addition of covariates, our moderation plots and conditional effects still suggest that exposure to enacted stigma plays a greater role in shaping TNBPOC people's bodily experience and health over the lifetime relative to white TNB people. The significant moderating effect of racial identity on *lifetime* enacted stigma but not *past year* enacted stigma suggests that these experiences accumulate over the life course.

Other research highlights how lived experiences of stigma may differ for TNBPOC people relative to white TNB people overall, leading to both increased resilience and risk. For example, Rood et al. (2016) highlight that TNBPOC people may feel better prepared to handle discrimination and rejection as a TNB person due to having managed previous experiences of racism. However, they also note that living as a TNBPOC person may also lead to increased experiences of discrimination and rejection overall due to collisions between systems of racism, cisnormativity, and gender binarism. Our findings align with this interpretation: compared to white TNB participants, at low amounts of lifetime enacted stigma, TNBPOC participants experienced less severe symptoms but at average and high levels of lifetime enacted stigma, TNBPOC participants experienced more severe symptoms.

While the differences in the associations between enacted stigma for TNBPOC and white TNB participants were relatively numerically small (e.g., 1–3 points), these small differences are meaningful on the human level. Here a 1‐point difference (e.g., the predicted difference at average amounts of lifetime enacted stigma differences between TNBPOC and white TNB people) is not only the difference between mild and moderate amounts of symptom severity per clinical cutoffs (9 vs. 10), but also represents one extra part of the body in distress or the difference between “a little” or a “a lot” of distress from that symptom. That extra distress, while numerically small, could have a large impact on that person's life, thus representing an important difference beyond what can be conveyed by statistical effect sizes alone.

Furthermore, the difference between our initial findings and our results related to past year and lifetime enacted stigma following the addition of covariates—chronic disease, in particular—suggests symptom severity is more explained by having a chronic disease among TNBPOC people in our study than among white participants. This may reflect several things. First, TNBPOC are more likely than white TNB people to have a diagnosed chronic disease (Seelman et al. 2017). Second, the experience of having a chronic disease is a racialized phenomenon; exposure to racism itself is associated with chronic health conditions (Lin 2025; Siddiqi et al. 2017), and thus may contribute as a driver of chronic diseases among TNBPOC relative to white TNB people. Despite this, POC in the United States are also more likely to have an *undiagnosed* chronic disease (Kim et al. 2018). So, in fact, the elevated rates of chronic disease among POC likely underreport the magnitude of inequity. Moreover, as a result of underdiagnosis and reduced access to treatment, all POC, regardless of gender/sex identities and experience, face heightened risk of morbidity and mortality from untreated chronic disease (Falagas et al. 2007), underscoring the need for further research in this area.

### 4.7 Queering Biocultural Studies of Health

Our work responds to the call to queer biocultural studies in two distinct ways: (1) we explicitly challenge taken‐for‐granted assumptions of the body as a cisgender heteronormative project purposefully disrupting expectations and norms (see, e.g., Halperin 1995; Kitzinger 1987; Seidman 1997). (2) By engaging with TNB bodies (broadly speaking, queer bodies), we explicitly critique heteronormative (and hegemonic) understandings of bodily symptoms and health (see, e.g., Hicks and Watson 2003; Zeeman et al. 2014). Our research exposes how the study of health and embodiment has contributed to dominant discourses of heteronormativity and in doing so directly challenges perduring cishet understandings of the body, embodied symptoms, and health more generally. Moreover, while the research described here engaged TNB individuals, our focus on biocultural measures of embodiment takes up Sedgwick's (1993) call to “spin the term [queer] outward along dimensions that can't be subsumed under gender and sexuality at all” (1993, 9). In other words, we reject that “queering” is reducible to TNB identities and following Eng et al. embrace the idea that there is not a fixed “subject of or object for” (Eng et al. 2005, 3) this approach. Indeed, investigating the health and biocultural embodiment of stress through a queer lens reveals the “biopolitical sorting of populations” specifically with regard to the severity of bodily symptoms (Eng and Puar 2020, 17).

Aligned with queer and feminist scholars' efforts to elaborate theory and methodologies to advance engagement with the complexities of sex and gender (e.g., DuBois et al. 2025; Pape et al. 2024; Smiley et al. 2024; Springer et al. 2012; Williams et al. 2023) as well as to recognize the lived entanglement of gender and sex, represented by the terms gender/sex and sex/gender (e.g., DuBois, Brewis, Burson, et al. 2024; Fausto‐Sterling 2019; Kaiser 2012; van Anders 2022), we aim to provide an example of how to study gender and sex beyond what categories can offer (see also DuBois, Puckett, Jolly, et al. 2024). In rejecting the either/or binary categories of sex and gender and instead using gender/sex (or sex/gender), we recognize the ways these categories are complex and entangled, refusing “categorical legibility” (Weiss 2022, 321). Relying on rigid binary categories also masks meaningful variation in human biology and reduces scientific accuracy by clouding the specific mechanisms through which differences arise (DuBois and Shattuck‐Heidorn 2021; Springer et al. 2012).

We approach gender and sex both as culturally co‐constructed and understood phenomena. As many feminist scholars have elaborated, the categorical labels for gender and sex, the cultural meanings and social roles associated with these categories, and the varied factors or components included in each of these categories are developed by people and thus are influenced by social norms within a given cultural context (Laqueur 1992; Markowitz 2024; Oudshoorn 1991). Spelling out how we are operationalizing gender and sex in research improves definitional precision and clarity in what exactly is being studied, thus contributing toward our understanding of the *mechanisms* underlying differences. In addition, it exposes unquestioned assumptions and cultural biases about gender and sex. Biocultural anthropologists aiming to model more inclusive data collection practices should be aware that not only do best language practices for capturing data about gender and sex rapidly change, but that the terms used in this study may not reflect current best practices for other cultural contexts (Dankwa 2021; Davis 2019; Shuster et al. 2024). Specific terminology used to capture information about gender and sex in research should always be informed directly by the communities and local contexts (DuBois, Brewis, Burson, et al. 2024).

A queering of embodiment and health demands a rethinking of how systems of oppression (e.g., cisnormativity and gender binarism) operate. Studying the lived experiences of enacted stigma, as we did, reveals how these oppressive systems affect TNB people's lives. For example, one of the experiences of anti‐TNB enacted stigma measured in this study is an inability to obtain identity documents that reflect participants' gender identities. This challenge is reflective of administrative processes that systematically delegitimize TNB people (thus reinforcing cisnormativity; Spade 2007). Issues such as this are particularly important in light of the current Trump–Vance administration's efforts to erase TNB people's ability to select a gender marker on federal documents (e.g., passports) that accurately reflects one's gender identity (Wong 2025).

This paper highlights how cisnormativity and gender/sex binarism become embodied and how and why using queer theory makes these issues visible. Our work thus disturbs (e.g., “queers”) dominant knowledge paradigms. It is important to note that “diversifying a sample” does not, on its own, disrupt or challenge assumptions or existing paradigms. Queering biocultural studies of health goes beyond simply including LGBTQIA+ people in research or replacing one set of assumptions with another (Seidman 1997). In fact, queer and feminist scholars compel us to center the role of *social power* in shaping health rather than focusing on identities alone (e.g., Bauer and Scheim 2019; Guan et al. 2021; Salem 2018). Here, we view embodiment as a conceptual framework to identify the experiential and physiologic pathways through which differences in social power lead to variation in health and well‐being (Krieger 1999, 2012). While we use moderation analyses to explore differences in social power, other methods are equally valuable to this end (e.g., Guan et al. 2021). Identification of these pathways gives us better targets for support and intervention, allowing us to move beyond simply identifying inequity toward eliminating inequity.

### 4.8 Future Directions and Limitations

While this study takes steps to identify how inequity influences embodied experiences among TNB people, this was a relatively small sample of TNB people in the United States, which limited our ability to test for interaction effects with enough power and precision to detect statistically significant results. Moreover, the effect sizes reported in this study are relatively small. Studies should be conducted among larger samples and in countries with varied sociopolitical contexts toward TNB people to see how results may change. These data are also cross‐sectional, which limits our ability to infer causal relationships. While we ground our interpretation in longitudinal studies of stigma and health among other marginalized groups (e.g., Sims et al. 2020), it is possible that the observed associations in our study reflect that people in our study reporting more severe symptoms may also be more likely to report enacted stigma and less resilience and support. Planned future analyses of longitudinal data from this study will help to elucidate the nature of these relationships.

Additionally, our questions did not explicitly specify whether we were asking about primary caregivers in the questions about “family” like we did with chosen family; thus, future research should include questions directly about primary caregivers and further include families beyond those conforming to traditional cisheteronormative structures. Cognitive interviews are needed to better understand how TNB people interpret “family” in measures that do not specify what family means.

As detailed in our discussion, another limitation reflects the need for more research on resilience among TNB people. This term is defined in many different ways in the literature and may also be conceptualized in marginalized communities in ways that go beyond the ability to simply “bounce back” (Puckett et al. 2022; Windle 2011). Others have critiqued resilience as a concept for overly focusing on an individual‐level trait and strategies (e.g., coping) that decontextualize larger social struggles, thus running the risk of reinforcing inequities in social power (Ojukwu et al. 2022; Schwarz 2018). Thus, there are likely other sources of resilience impacting health among TNB people that are not captured by this narrower definition of resilience (Puckett, Kimball, et al. 2024). Future research investigating other forms and meanings of resilience among TNB people and the effects on health and wellbeing would be most valuable.

We were also limited in our ability to determine the exact mechanisms driving differences in embodied experience by gender and racial identity. Qualitative research is needed to better understand what influences health among nonbinary people and TNBPOC to better capture differences in lived experience beyond the capacity of current scales. Our results also suggest a need for research with TNBPOC that adopts an explicitly intersectional perspective that explores how differences in proximity to social power influence structural, interpersonal, and individual factors that shape TNBPOC's health over the lifetime. This study was also limited in its ability to parse how lived experiences vary among the TNBPOC participants, as the relatively limited racial and ethnic diversity in this sample led us to collapse experiences of all TNBPOC into a singular group to maximize analytic power. Further research is needed that separates specific racial groups rather than collapsing all racial minorities into one group of TNBPOC, as well as research that disrupts the norm of whiteness as the standard against which all other groups are compared.

Finally, while here we focus on symptom severity, it is equally important to focus on *positive* lived experiences of the body, such as joy, thriving, and pleasure. By doing so, we can challenge deficit frameworks and paradigms that treat those whose social identities and positions fall outside of restrictive norms as inherently deficient and disordered (Bryant et al. 2021). Toward this end, a growing body of scholars in TNB health now recognize the importance of gender euphoria—positive feelings of comfort, joy, relief, and satisfaction when one feels affirmed by their lived experiences of gender (Austin et al. 2022; Beischel et al. 2021; Jacobsen and Devor 2022; Jones 2023; Skelton et al. 2023).

## Author Contributions

**Dee Jolly:** formal analysis (lead), methodology (equal), visualization (lead), writing – original draft (lead), writing – review and editing (equal). **Jae A. Puckett:** conceptualization (equal), formal analysis (supporting), investigation (equal), methodology (equal), project administration (equal), supervision (equal), writing – original draft (supporting), writing – review and editing (equal). **Cindi SturtzSreetharan:** writing – review and editing (equal). **Sawyer E. Armstrong:** writing – original draft (supporting), writing – review and editing (equal). **Debra A. Hope:** investigation (equal), supervision (equal), writing – review and editing (equal). **Richard Mocarski:** investigation (equal), supervision (equal), writing – review and editing (equal). **Robert‐Paul Juster:** investigation (equal), writing – review and editing (equal). **L. Zachary DuBois:** conceptualization (equal), investigation (equal), methodology (equal), project administration (equal), supervision (equal), writing – original draft (lead), writing – review and editing (equal).

## Data Availability

The data for this study are not publicly available due to privacy and ethical restrictions.
